# Risky Decision-Making in Schizophrenia: Examination of association between smoking, substance use and performance on the Iowa Gambling test: Pilot Study

**DOI:** 10.1192/j.eurpsy.2022.1987

**Published:** 2022-09-01

**Authors:** P. Dannon, S. Kertzman

**Affiliations:** Tel Aviv University, Psychiatry, tel aviv, Israel

**Keywords:** substance use, decision-making, schizophrénia, nicotine

## Abstract

**Introduction:**

There are two common hypotheses to explain such high comorbidity between nicotine dependence and schizophrenia (SZ): self-medication for decreasing psychiatric symptoms or common environmental risk factors can predispose to both nicotine dependence and other risky behaviors in SZ

**Objectives:**

Little is known about the influence of cigarette smoking comorbidities such substance use disorder (SUD), criminal history, or risky decision among patients with SZ.

**Methods:**

The Iowa Gambling test (IGT) was administered to thirty-nine patients with SZ of whom 69% reporting cigarette smoking. Both groups were evaluated using a socio-demographic questionnaire and clinical assessment using PANSS and self-report questionnaire the Barratt Impulsiveness Scale (BIS-11). To evaluate decision making was evaluated with the Iowa Gambling Task (IGT).

**Results:**

The full SZ sample performed worse on the IGT then normal population. Smokers with SZ performed significantly worse than nonsmokers on the IGT primarily because they preferred “disadvantageous” decks to a greater degree. The PANSS and impulsivity tendencies (BIS-11) did not predict overall performance on the IGT. Smokers with SZ had impaired affective decision-making. Behavior suggested preferential attention to the frequency amount of gain and inattention to amount of loss suggesting impairments in risk/reward decision-making

**Conclusions:**

This study is the first to compare IGT in smokers and nonsmokers with SZ with adjustment of SUD, criminal history, and existing tattoo to further examine IGT performance. These results support the hypothesis that comorbidities between nicotine dependence and SZ can be linked to other common factor that is associated with other externalizing behaviors in SZ.

**Disclosure:**

No significant relationships.

